# Sustained DMARD-free remission in rheumatoid arthritis − about concepts and moving towards practice

**DOI:** 10.1016/j.jbspin.2022.105418

**Published:** 2022-05-27

**Authors:** Marloes Verstappen, Annette H.M. van der Helm-van Mil

**Affiliations:** a Department of Rheumatology, Leiden University Medical Centre, Leiden, The Netherlands; b Department of Rheumatology, Erasmus Medical Centre, Rotterdam, The Netherlands

**Keywords:** Rheumatoid arthritis, Auto-antibodies, DMARD-free remission, Drug-free remission

## Abstract

Sustained DMARD-free remission (SDFR) is the best possible outcome in RA. It is characterized by sustained absence of clinical arthritis, which is accompanied by resolution of symptoms and restoration of normal physical functioning. Therefore it’s the best proxy for cure in RA. The mechanisms underlying SDFR-development are yet unidentified. Hypothetically, there are two possible scenarios. The first hypothesis is based on the concept of regaining immune-tolerance, which implies that RA-patients are similar at diagnosis and that disease-processes during the disease-course shift into a favorable direction, resulting in regaining a state in which arthritis is persistently absent. This could imply that SDFR is theoretically achievable for all RA-patients. The alternative hypothesis is that RA-patients who achieve SDFR are intrinsically different from those who cannot. This would imply that DMARD-cessation could be restricted to a subgroup of RA-patients. Since the 1990s, DMARD-discontinuation and SDFR have been increasingly studied as long-term-outcome in RA. In this review, we describe hitherto results of clinical, genetic, serological, histological and imaging studies and looked for arguments for the first or second hypothesis in both auto-antibody-positive and auto-antibody-negative RA. In auto-antibody-negative RA, SDFR is pre-sumably restricted to a subgroup of patients with high serological-markers of inflammation at diagnosis and a rapid and sustained decrease in inflammation after treatment-start. Identifying these RA-patients could be helpful in realizing personalized-medicine. In auto-antibody-positive RA, only few patients achieve SDFR and no definite conclusions can be drawn, but data could suggest that SDFR-patients might be a subgroup with relatively low inflammation from disease-presentation onwards.

## 1 Introduction

Sustained DMARD-free remission (SDFR), the sustained absence of clinical arthritis after discontinuation of all DMARD-therapy, is increasingly achievable in rheumatoid arthritis (RA) [1,2]. As SDFR is accompanied by resolution of symptoms like pain and fatigue, and by restoration of normal physical functioning, it can be regarded the best proxy for cure in RA [2]. Nevertheless, mechanisms underlying SDFR-development are poorly understood and the concept of ‘cure’ contrasts the general perception that RA is a chronic disease requiring life-long disease-modifying treatment. In this review, we propose two concepts that may explain disease-resolution in RA. We evaluated hitherto scientific evidence of clinical, serological, histological, imaging and genetic studies on SDFR in RA and studied these to substantiate the two hypotheses, with the ultimate aim to increase understanding of permanent disease-resolution in autoantibody-positive and autoantibody-negative RA.

## 2 Two concepts to understand SDFR-development: regaining-immune-tolerance-hypothesis versus subgroup-hypothesis

**Fig. 1:**
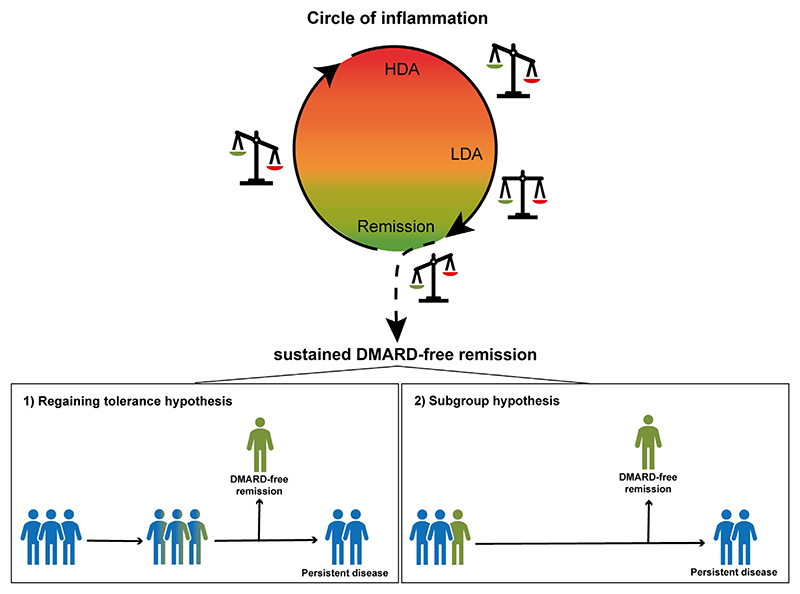
Hypotheses which could explain sustained DMARD-free remission in rheumatoid arthritis. RA-patients achieving sustained DMARD-free remission escape the ongoing process of inflammation. For disease-resolution, it could either be proposed that processes shift into a favorable direction in which markers of disease chronicity − yet unidentified − are lost over time, allowing to achieve SDFR (hypothesis 1). Or, SDFR could be determined by specific characteristics of a subgroup of RA-patients; then patient achieving SFDR are intrinsically different (hypothesis 2). LDA: low disease activity; HDA: high disease activity; DMARD: disease-modifying anti-rheumatic drugs; RA: rheumatoid arthritis.

The first hypothesis is based on the concept of regaining immune-tolerance. This is based on the notion that RA-development is a multiple-hit process in which, at a certain point in time, immune-tolerance is lost and disease-chronicity established. For disease-resolution, processes shift into the opposite, favorable direction. Biological markers of disease chronicity − yet unidentified − are lost over time, allowing to achieve SDFR. This hypothesis implies that RA-patients are similar at diagnosis and that differences may emerge over time (Fig. 1). Alternatively, it can be hypothesized that RA-patients achieving SDFR are intrinsically different from RA-patients who cannot achieve SDFR. In this scenario, RA-patients achieving SDFR carry favorable characteristics due to which the disease is more prone to resolve. This would imply that once known which markers characterize this subgroup, patients that can achieve SDFR could be identified already at diagnosis and treatment-decisions could be personalized early in the disease (Fig. 1). A difference between the two hypotheses is that in the first hypothesis the likelihood of achieving SDFR is more or less equal for RA-patients at diagnosis and differentiation occurs over time, whereas hypothesis 2 assumes that the differences in the likelihood of SDFR are already determined at disease-presentation or very early in the disease.

## 3 SDFR definition is essential to ensure sustainability

**Fig. 2:**
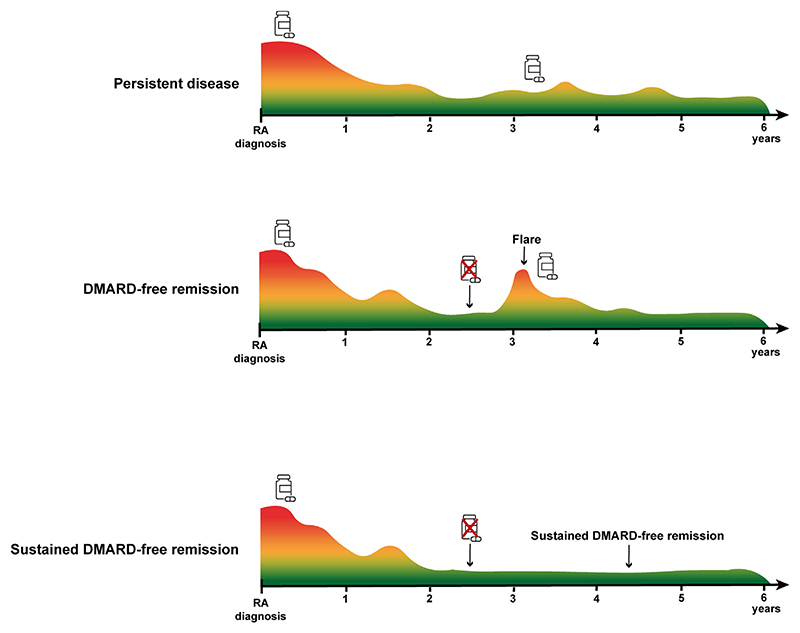
Disease course of rheumatoid arthritis and subsequent development of (sustained) DMARD-free remission. RA-patients could either have persistent disease (upper timeline) or achieve remission and are subsequently able to discontinue their DMARD-therapy (lower two timelines). The middle timeline illustrates the situation in which RA-patients were able to discontinue their DMARD-treatment but were not able to maintain remission after DMARD-stop. In the lower timeline, patients were not only able to discontinue their DMARD-treatment but were also able to sustain remission for minimally one year after DMARD-stop (and the subsequent follow-up thereafter).

Although DMARD-discontinuation in RA has been studied as early as the 1990s, definitions for DMARD-free remission (DFR) vary widely between studies and several different remission-measures have been used to define DMARD-free remission; e.g. DAS, CDAI, SDAI or Boolean remission [3]. Some of these measures have been criticized, as they allow a certain level of residual inflammation which is not in line with the concept that DFR is a proxy for cure [4]. A strict definition, allowing no clinical signs of disease activity, is therefore preferred when defining DFR. Moreover, there is a distinct difference between “DFR” and “sustained DFR” (SDFR) [3]. DFR refers to the state in which RA-patients are able to discontinue their DMARD-treatment but does not impose a duration in which remission is maintained after DMARD-stop (Fig. 2). A recent systematic literature review demonstrated that most flares occur in the first-year after DMARD-cessation; the flare rate after successful DMARD-tapering was on average 45–50% in the first year, whilst this significantly decreased to 5–18% after the first year [3]. Hence, achieving DFR is a status that does not provide information on sustainability (Fig. 2). In order to approximate cure, the sustainability of remission needs to be ensured. There is no consensus on which duration of remission is sufficient to define this sustainability. However, since it has been shown that flares are less frequent once remission has been maintained for minimally 1-year after DMARD-cessation [3], a minimal time-frame of at least 1-year in the definition of SDFR seems warranted. Several (observational) studies described that SDFR was achieved approximately 3–4 years after disease-presentation, which means that DMARDs were discontinued 2–3 years after DMARD-initiation (Fig. 2).

In this review, we specifically focus on SDFR, i.e. sustained remission for minimally 1-year after complete DMARD- discontinuation (including corticosteroids). A systematic literature- search was carried out in PubMed to identify studies reporting on SDFR in RA (see Online material supplementary S1 for used terms). References of these studies were used to find additional studies by hand-search. Of the 141 identified studies, 81 reported on DMARD-discontinuation in RA of which 39 studies reported on sustained DMARD-free remission (Online material supplementary S2). Clinical, serological, imaging, histologic and genetic factors studied in these articles in relation to SDFR are discussed here and reviewed in the light of the two hypotheses. Identified factors were stratified for the moment they were measured: at baseline (Online material supplementary S3), during the disease course (Online material supplementary S4) or at the moment of DMARD-discontinuation (Online material supplementary S5).

## 4 Autoantibody positive and negative RA: two disease subsets

Growing evidence supports the idea that RA can be classified into two distinct disease-entities; auto-antibody-positive and auto-antibody-negative-RA [1,5]. This concept is based on the multiple differences found between auto-antibody-positive and auto-antibody-negative RA-patients regarding their genetic background [6], environmental risk-factors [7,8], preclinical symptomatic-phase [9], clinical disease-presentation [9–11], and different immuno-histological characteristics [12,13]. Importantly, also long-term outcomes, like mortality and radiographic-progression [14,15], diverge widely between auto-antibody-positive and auto-antibody-negative RA [1]. Likewise, SDFR-prevalence differs significantly between these RA-subsets, in which SDFR is frequently achieved in auto-antibody-negative RA (40%) but rarely in auto-antibody-positive RA (5–10%) [1]. Since mechanisms underlying disease-resolution might also be inherently different in auto-antibody-positive and auto-antibody-negative RA, we will evaluate all scientific data on SDFR-development separately for auto-antibody-positive and auto-antibody-negative RA-patients to explore whether different hypotheses might account for disease-resolution.

## 5 Genetic factors associated with SDFR

Genetic-variants have been linked to RA-susceptibility and disease-severity and associations have been observed with radiological-damage and mortality in RA [16–21]. If specific genetic-variants can determine a trait which makes SDFR-development more accessible in RA-patients with these variants, this would naturally fit the subgroup-hypothesis as genetic-variation is determined long before disease-onset. However, it can also be proposed that specific genetic-domains contribute to more general processes leading towards restoration of immune-tolerance once markers of chronicity are turned off over time. Of the 5 genetic studies published with SDFR as outcome (Online material supplementary S3) [22–26], 3 studies focused on shared epitope (SE) which presence was associated with a decreased risk of SDFR [22–26]. However, this association disappeared after correction for ACPA-positivity, suggesting that SE and ACPA are part of the same pathway towards SDFR. One study also evaluated other genes in relation to SDFR; nine radiographic-progression-associated genetic variants (*SE* in *HLA-DRB1, rs1896368/rs1896367/rs1528873* in *Dickkopf-1, rs2104286* in *IL2RA, rs26232* in C5Orf30, *rs11908352* in *MMP-9, rs451066* at chromosome 14, and *rs1485305* in osteoprotegerin), and was auto-antibody-stratified [25].

**Fig. 3:**
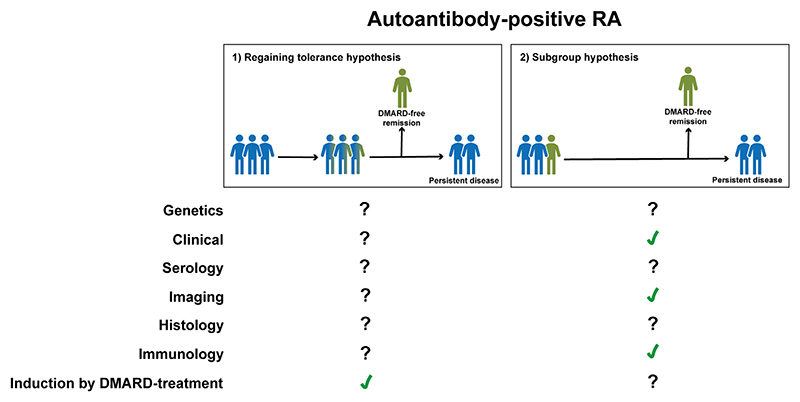
Scientific substantiation for both hypotheses to understand disease-resolution in auto-antibody-positive-RA. Scientific evidence on biomarkers related to SDFR in auto-antibody-positive RA, summarized from the perspective of both hypotheses. A tick symbol indicates that findings on this topic are suggestive for the specific hypothesis. Crosses indicate that findings are suggestive that the hypothesis is not plausible. A question mark indicates that there is no data on this topic which can be related to one of the two hypotheses.

In auto-antibody-positive RA, none of the 9 genes were associated with SDFR-development, although the number of patients achieving SDFR was low which limited statistical-power (Fig. 3) [25]. Interestingly, in auto-antibody-positive RA, shared epitope was not independently associated with SDFR which suggests that SE might have no other role in disease-persistency than via ACPA.

**Fig. 4:**
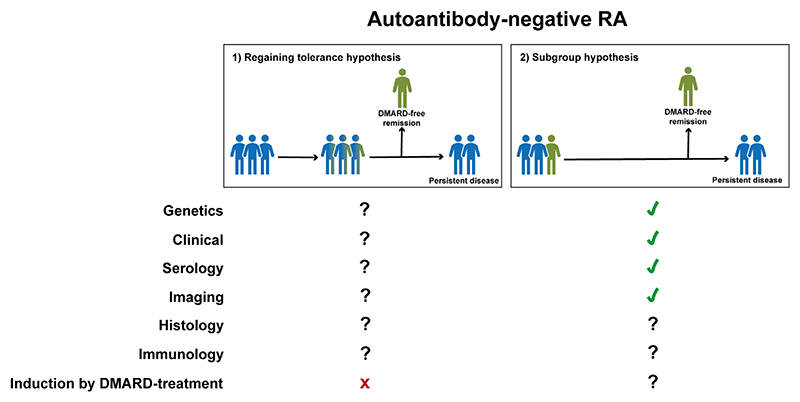
Scientific substantiation for both hypotheses to understand disease-resolution in auto-antibody-negative-RA. Scientific evidence on biomarkers related to SDFR in auto-antibody-negative RA, summarized from the perspective of both hypotheses. A tick symbol indicates that findings on this topic are suggestive for the specific hypothesis. Crosses indicate that findings are suggestive that the hypothesis is not plausible. A question mark indicates that there is no data on this topic which can be related to one of the two hypotheses.

In auto-antibody-negative RA, specific genetic-variance of *IL2RA (rs2104286)* was associated with increased SDFR-prevalence [25]. *IL2RA* encodes the *α*-chain of the high-affinity IL-2 receptor, which is involved in homeostasis and function of regulatory T-cells which in turn are essential for self-tolerance [27]. This specific *IL2RA*-variant also associates with lower IL-2 serum-levels, and lower IL-2 serum-levels during the disease-course were also associated with more SDFR. Genetic variants in *IL2RA* were still significantly associated with SDFR after correction of serum-Il-2 levels, suggesting that part of its effect exerts via a different pathway than via serum IL-2-levels. Since RA-patients who did not have this *IL2RA*-variant achieved SDFR less often, the subgroup-hypothesis may be felt most plausible here (Fig. 4). Since most genes that conferred risk for radiographic-damage genes were not associated with SDFR, mechanisms driving joint-damage progression and disease-persistence might be mostly different.

## 6 Clinical characteristics associated with SDFR

When specific clinical characteristics can be linked to SDFR-development this will not only contribute to further understanding of disease-resolution in RA, but it makes personalization of DMARD-tapering more accessible for clinical practice. As several patient characteristics (age, gender, BMI) were not associated with SDFR, studies focused on more disease-specific clinical characteristics like disease-activity, symptom-duration and physical functioning (Online material supplementary S3). Intuitively, SDFR might appear more likely in RA-patients with less clinical inflammation (less involved joints, lower acute-phase reactants) at disease-presentation and in those who attain clinical remission during DMARD-treatment, as switches of chronicity might be more easily turned off and immune-tolerance regained. However, we know that not all RA-patients in clinical remission can successfully taper their DMARD-treatment and other patient-characteristics seem required to attain SDFR (subgroup-hypothesis). In total, 19 articles reported on the relation between various clinical-markers and SDFR in the total RA-population (Online material supplementary S2). Although a wide range of clinical-characteristics at disease-presentation was studied, results appeared conflicting (Online material supplementary S3). With a lack of association between baseline clinical-characteristics and SDFR, also clinical markers during the disease course (Online material supplementary S4) and at time of DMARD-discontinuation have been studied (Online material supplementary S5) [28,29]. Only four studies were stratified for the presence of auto-antibodies (1 study under-review) [28,30–32].

In auto-antibody-positive RA, a recent study in two separate auto-antibody-positive RA-populations reported that patients achieving SDFR had lower disease-activity-scores (DAS) and less swollen-joints at disease-presentation (data under review). In one of these cohorts, not only baseline-SJC was lower, but also the other individual DAS-components (CRP/TJC/global health). A study focusing on DAS-trajectories after DMARD-initiation, demonstrated that subsequent DAS-trajectories during follow-up were not different in patients achieving SDFR, but statistical power was limited since only few auto-antibody-positive RA-patients achieved SDFR [28]. The finding that auto-antibody-positive RA-patients achieving SDFR have already less inflammation from disease-presentation onwards might intuitively suggest that the inflammatory response in these patients is less developed and therefore more easily suppressed by DMARD-treatment after which immune-tolerance may be easier restored. If so, a shorter symptom-duration in SDFR-patients would be expected. Although one study reported that a shorter symptom-duration was associated with SDFR [31], this was not replicated [33]. Thus, that auto-antibody-positive RA-patients achieving SDFR have a lower disease activity already from diagnoses onwards seems to be better explained by the subgroup-hypothesis (Fig. 3).

In auto-antibody-negative RA, no differences were found in baseline DAS, nor in the individual DAS-components (SJC/TJC/ESR/GPA) at baseline [28]. However, auto-antibody-negative RA-patients achieving SDFR did have a significantly stronger decline in DAS in the first 4-months of DMARD-treatment [28]. In patients with early DAS-remission (DAS < 1.6 after 4-months) the likelihood of achieving SDFR increased up to 75%, compared to 40% in other auto-antibody-negative RA-patients. Of the DAS-components, predominantly SJC and ESR declined stronger in SDFR-patients. Moreover, higher baseline CRP-levels are associated with SDFR in auto-antibody-negative-RA [30,32], which might appear counterintuitive. However, a follow-up study demonstrated that auto-antibody-negative RA-patients with high baseline CRP-levels also have a subsequent stronger decline in CRP-levels during the first year of DMARD-treatment, stronger than patients who do not achieve SDFR [30]. Thus, patients achieving SDFR are characterized by high baseline CRP-levels and a subsequent strong decline in DAS and CRP upon DMARD-initiation. The combination of both high baseline CRP-levels and early DAS-remission even resulted in 85% SDFR, suggesting that patients who share these favorable characteristics define a subgroup of patients most likely to attain disease-resolution (Fig. 4).

## 7 Serologic inflammation markers associated with SDFR

Next to the commonly used (non-specific) inflammatory-markers CRP and ESR, other, more disease-specific, systemic (inflammatory) markers, might be informative to understand disease-resolution in RA. Two serological biomarker- studies, focusing on 12 serum-protein-biomarkers (MMP-1/MMP-3/SAA/CRP/ IL-6/leptin/resistin/YKL-40/TNF-R1/EGF/VEGF/VCAM-1) related to systemic-inflammation and bone-degradation, were auto-antibody-stratified [30,32].

In auto-antibody-positive RA-patients in both studies, neither baseline levels of these biomarkers, nor the change in levels of these markers in the first 2-years of DMARD-treatment, were associated with later on development of SDFR (Fig. 3) [30,32]. Yet, the number of auto-antibody-positive-RA-patients achieving SDFR was low, limiting statistical-power.

In auto-antibody-negative RA, one study demonstrated that auto-antibody-negative-RA-patients achieving SDFR had not only higher baseline CRP-levels, but also baseline levels of MMP-3 and SAA were higher [32]. Although also this initially appeared counterintuitive from a disease-resolution perspective, also here a follow-up study demonstrated that the levels of these serological markers (and MMP-1) declined significantly stronger in the first year of DMARD-treatment in auto-antibody-negative RA-patients achieving SDFR [30]. Interestingly, this decline in serological levels in the first year was strongest in SDFR-patients achieving early DAS-remission, suggesting that the combination of these favorable patient-characteristics define a subgroup most likely to achieve SDFR (Fig. 4).

## 8 Imaging findings associated with SDFR

Advanced imaging-modalities have contributed significantly to the understanding of tissues inflamed in RA [34–36]. Whereas conventional radiographs can only visualize erosive changes, ultrasound and MRI are sensitive measures of local joint-inflammation in synovium, tenosynovium and (for MRI) bone [37]. This allows to study joint-inflammation in more detail in relation to SDFR-development.

No ultrasound studies have been conducted in relation to SDFR in RA. Of the 6 imaging-studies [22,23,33,38], two studies that used MRI of hand and foot-joints were auto-antibody-stratified (1 under review) [33].

In auto-antibody-positive RA, less MRI-detected joint-inflammation (especially synovitis and osteitis) and erosions at diagnosis, and during subsequent follow-up, were associated with SDFR (Online material supplementary S3 and S4) [39]. This was validated in an external auto-antibody-positive RA-population [39]. Subsequently, another study linked less MRI-detected synovitis and erosions at time of DMARD-discontinuation to more SDFR (Online material supplementary S5) [33]. Thus, also here auto-antibody-positive RA-patients achieving SDFR had significantly less joint-inflammation from disease-presentation onwards. Importantly, the symptom duration was not shorter in patients that later achieved SFDR, suggesting that the lower levels of inflammation may be a patient-characteristic (subgroup-hypothesis, Fig. 3). Nevertheless, definite conclusions should not be drawn yet because of low number of auto-antibody-positive RA-patients achieved SDFR in these analyses.

In auto-antibody-negative RA, MRI-detected joint-inflammation did not differ at baseline between patients who did and did not achieve SDFR. However, the decline in MRI-detected inflammation (especially tenosynovitis and osteitis) in the first-year of DMARD-treatment was significantly stronger in patients achieving SDFR [39]. Synovitis, tenosynovitis and osteitis declined together, in contrast to auto-antibody-positive RA in which tenosynovitis did not play a role in SDFR. Thus, auto-antibody-negative RA-patients achieving SDFR are comparable to other RA-patients at baseline but demonstrate a stronger decline in inflammation (measured with the DAS, serological marker and imaging of the joints) after treatment-start. This suggests that underlying disease-processes might be inherently different and respond differently to DMARD-therapy which in turn appears favorable for attaining disease-resolution (subgroup-hypothesis, Fig. 4).

## 9 Histological characteristics associated with SDFR

Histological studies can reveal fundamental processes underling RA-chronicity and are therefore of great interest when aiming to understand disease-resolution. Despite extensive histological studies on the pro-inflammatory processes in RA, histological studies on disease-remission are scarce [40]. Yet, it would be of interest whether local (inflammatory) processes are inherently different from diagnosis onwards in patients achieving SDFR (subgroup-hypothesis) or whether normal joint-homeostasis can theoretically be restored in all RA-patients when disease-processes shift into a favorable direction (regaining immune-tolerance-hypothesis). Histological studies on SDFR in RA are absent. However, an interesting histologic study was published recently about RA-patients who were in clinical remission after DMARD-tapering [40]. Using single-cell-sequencing of synovial tissue, synovial tissue macrophages (STMs) were identified which appeared to play a role in regulating joint inflammation. MerTK(pos) STMs were high in RA-patients in remission and low in active disease; and a low proportion of MerTK(pos) STMs in RA-patients in remission was associated with an increased risk of flare after DMARD-tapering. However, since histologic or molecular-studies during the development of SDFR are absent, it remains to be determined whether such innate cells in the joint tissue are involved in SDFR-development.

## 10 Auto-antibody response characteristics

Several different auto-antibodies, e.g. RF, ACPA, anticarbamylated protein (anti-CarP), anit-acetylated protein antibodies (AAPA), have been studied in relation to SDFR [41–43]; but only the presence of ACPA appeared to be independently associated with SDFR, irrespective of the co-existence of other auto-antibodies (Online material supplementary S3) [41,43]. Nonetheless, it remains unclear whether ACPA plays an active role in persistent disease, or whether ACPA is solely a bystander [44]. ACPA can be present for years before the disease-onset and maturation of the ACPA-response appears to occur predominantly in the asymptomatic pre-arthritis phase of RA, and according to current knowledge is not the final step towards developing RA [45,46]. In addition, in the pre-RA-phase of clinically-suspect-arthralgia, a matured auto-antibody-response also occurs in ACPA-positive patients who do not transition to RA but achieve resolution [45]. The question is to what extent ACPA-characteristics are related to achieving resolution of RA. It has been demonstrated that ACPA-levels are not associated with SDFR [47,48]. Moreover, it has been hypothesized that the disappearance of auto-antibodies, defined as ¨immunological remission¨, would occur before or at the time that disease-resolution is achieved [49]. However, interestingly enough, it has been demonstrated that SDFR is not paralleled by the disappearance of ACPA or by a reduction in ACPA-levels [47]. Also ACPA-IgM remained similarly present in RA-patients achieving SDFR, showing a disconnection between the ACPA-response and a beneficial clinical disease-course [38]. Further studies are needed to learn how “immunological-remission” should be defined in order to explain disease-resolution in RA on immunological-level. Interestingly, whereas extensive glycosylation of the variable (Fab) domain is a remarkable molecular-feature of ACPA-IgG, a recent study demonstrated that levels of ACPA Fab-glycosylation are significantly lower at disease-onset in ACPA-positive RA-patients achieving SDFR, compared to age- and gender-matched RA-patients not achieving SDFR [46]. Since these differences in auto-antibody-characteristics were already present at disease-presentation, it appears suggestive for a subgroup of auto-antibody-positive RA-patients in whom RA more easily resolves (Fig. 3), rather than a change in auto-antibody-characteristics over time that relates to regaining immune-tolerance. However, numbers of auto-antibody-positive patients achieving SDFR were low and further studies are warranted.

## 11 Treatment & SDFR: can we induce SDFR with disease-modifying treatment?

The ultimate question would be whether we can induce disease-resolution in RA, thus are we able to actively promote cure of RA? If disease-resolution can be explained with the subgroup-hypothesis, SDFR is reserved for only a part of the RA-population and cannot be induced in the rest. Identification of this subgroup at disease-presentation or early in the disease can lead to personalized treatment-decisions to taper DMARDs once remission is achieved. However, from the perspective of the regaining-immune-tolerance hypothesis, if SDFR would be inducible, this could theoretically be possible in all RA-patients. Thus, understanding SDFR from the perspective of these hypotheses is essential for future management of RA. Several studies have explored whether different treatment-strategies led to higher prevalence of SDFR in RA (Online material supplementary S6). Five of these studies were stratified for auto-antibodies [1,2,23,33,50].

In auto-antibody-positive RA, SDFR-prevalence was higher among patients undergoing DAS-driven-therapy compared to those receiving non-DAS-driven therapy [23,50]. In turn, treatment-steering at DAS < 1.6 led to more SDFR than treatment-steering at DAS < 2.4 [50]. In line with this, a more recent study demonstrated that early and intensive DMARD-treatment led to a higher incidence of SDFR in auto-antibody-positive RA [14]. This suggests that SDFR might be inducible in this patient-population, which fits the regaining immune-tolerance hypothesis (Fig. 3). Nevertheless, SDFR is still infrequent in auto-antibody-positive RA and future studies have to point out whether these treatment-effects are present in all auto-antibody-positive patients or confined to a subgroup of patients (for instance those presenting with less severe inflammation or less F(ab)-glycosylation of ACPA).

In auto-antibody-negative RA, the transition to early and intensive treatment-strategies over time did not increase SDFR-prevalence [14,50], which suggest that achieving SDFR is determined by patient-characteristics rather than treatment-strategies which support the subgroup-hypothesis (Fig. 4).

## 12 Discussion

In auto-antibody-positive RA, SDFR is infrequent which limited statistical-power to characterize these patients. Nevertheless, the available data could suggest that auto-antibody-positive RA-patients achieving SDFR represent of subgroup of patients with specific characteristics at baseline (Fig. 3); less clinically apparent and MRI-detected joint-inflammation and different auto-antibody-characteristics (less ACPA Fab-glycosylation). However, the fact that more intensive DMARD-strategies have increased SDFR-prevalence in auto-antibody-positive-RA could also support the regaining-immune-tolerance hypothesis. Future studies on SDFR in auto-antibody-positive-RA, ideally with a larger number of patients achieving SDFR, remain warranted to draw more definite conclusions.

In auto-antibody-negative RA, patients achieving SDFR are characterized by a combined strong decline in both clinical inflammation, serological inflammatory-markers and MRI-detected joint-inflammation in the first year of DMARD-treatment. These findings suggest that auto-antibody-negative RA-patients achieving SDFR represent a subgroup with favorable characteristics leading to a relatively early and adequate response to DMARD-treatment and subsequent disease-resolution later on (Fig. 4). Since enhanced DMARD-strategies did not increase SDFR-prevalence in auto-antibody-negative RA, the regaining immune-tolerance hypothesis is less likely. Future research might be able to further characterize this subgroup of patients in order to personalize treatment-decisions. set as new para here Ä limitation of this study..Ä limitation of this study is that this was not a systematic literature review. The literature was assessed semi-systematically due to which certain SDFR-studies might have been missed. Although multiple articles were identified, several reported on the same patient-populations, limiting the variance in the data. Most studies were observational by design and had sufficient follow-up to ensure sustainability of the outcome, which is a strength. A disadvantage of observational studies is that tapering was non-protocolized and based on shared-decision making between patients and clinicians. Although this resembles routine-care, patients who were in remission but did not taper their DMARD-treatment could have underestimated the SDFR-population. Trials in cooperating protocolized DMARD-discontinuation and a sufficient follow-up would be preferable, however these studies are costly. Moreover, although we studied the effect of more intensive DMARD-treatment on SDFR, we were not able to determine whether a specific type of DMARD is more favorable for achieving SDFR as this data was limited.

In this review, we used a strict SDFR-definition, including remission for minimally 1-year after DMARD-stop in order to ensure sustainability and to approximate cure. Nevertheless, we did not restrict on remission-definition as this would limit the number of eligible studies. Although most studies used a strict remission-definition, some studies used DAS-remission, which allows some residual inflammation. Consensus on a strict remission-definition is required for future studies on SDFR in RA.

We proposed two hypotheses in order to explain disease-resolution in both auto-antibody-positive and auto-antibody-negative RA. As pathological mechanisms in RA are complex and multifactorial [5,51,52], the proposed hypotheses might be somewhat simplistic. Although this was helpful in summarizing current evidence on disease-resolution in RA, there might also be other hypotheses which could explain disease-resolution and which were not included here.

This is the first review with combined evidence from multiple studies and that suggested that mechanisms of disease-resolution might be inherently different in auto-antibody-positive and auto-antibody-negative RA. So far, only few studies stratified for auto-antibodies and future research on processes underlying achieving SDFR would benefit from stratification for auto-antibody-status.

## Supplementary Material

S1, S2, S3, S4, S5, S6

## Data Availability

All data relevant to the study are included in the article or uploaded as supplementary information. Additional data are available upon reasonable request.
