# Supplementary material for: Sustained DMARD-free remission in rheumatoid arthritis − about concepts and moving towards practice
Source: Joint Bone Spine. Author manuscript; Available in PMC 2024 Apr 25. (PMC7615888; doi:10.1016/j.jbspin.2022.105418)
Supplement: S1, S2, S3, S4, S5, S6 [file EMS195436-supplement-S1__S2__S3__S4__S5__S6.docx]

**Supplementary S1. PubMed search and selection of eligible studies**

**PubMed search:** (rheumatoid arthritis) AND ((drug-free remission) OR (DMARD-free remission))

Date of search:

**Results: 141**

##
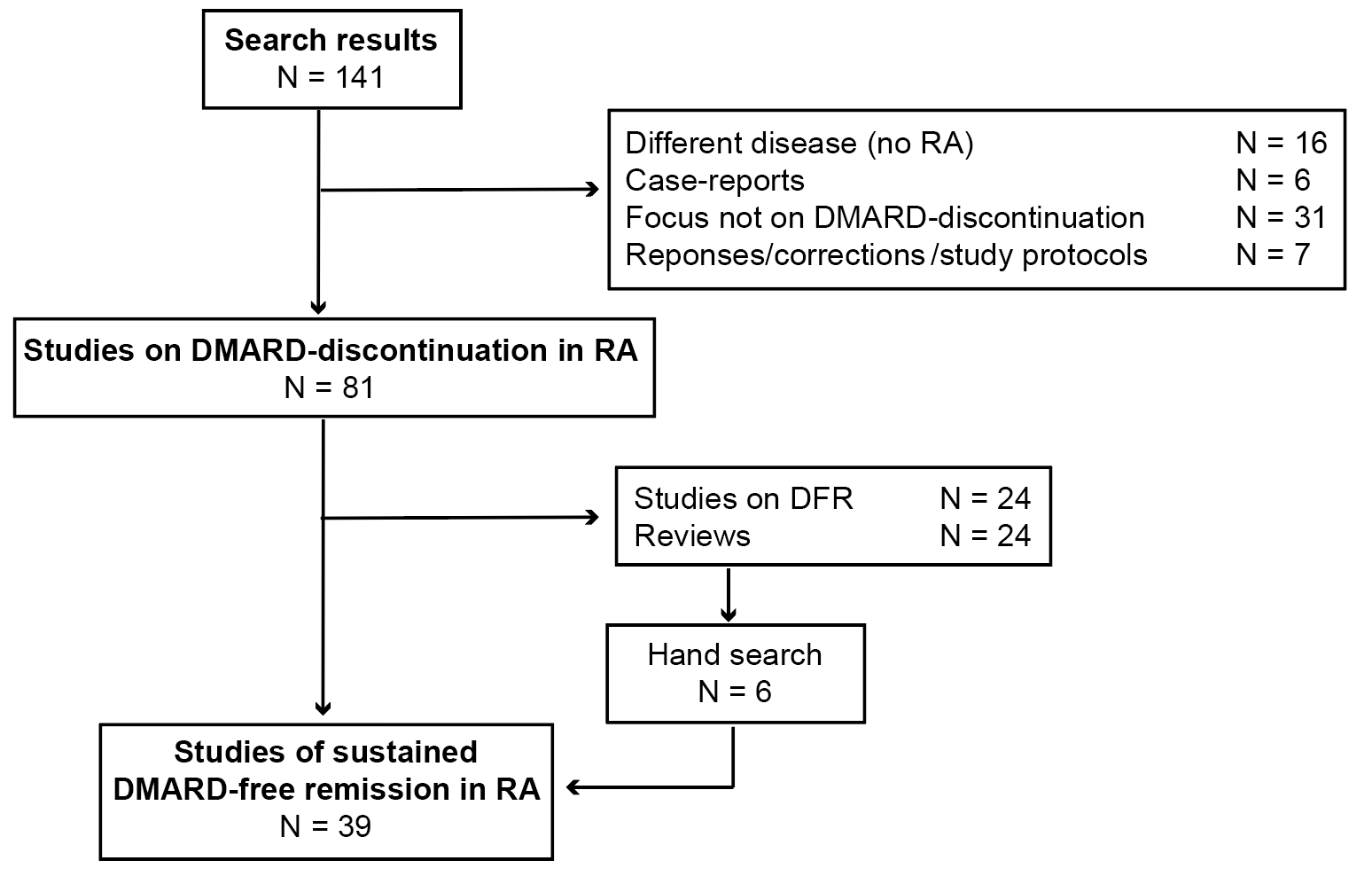


**Legend:** Flowchart of studies resulting from the systematic literature search in PubMed.

*RA: rheumatoid arthritis, DFR: DMARD-free remission, SDFR: sustained DMARD-free remission*

**Supplementary S2. Identified studies reporting on DMARD-discontinuation in rheumatoid arthritis**

| **Author (year)** | **Cohort** | | | **Remission definition after DMARD-stop** | | **Remission**  **duration after DMARD-stop** | **SDFR** | **Clinical** | **Serology** | **Imaging** | **Histology** | **Genetics** | **Autoantibodies** | **Treatment** | **Moment markers were measured** |
| --- | --- | --- | --- | --- | --- | --- | --- | --- | --- | --- | --- | --- | --- | --- | --- |
| Kissel et al. (2022)[1] | LEAC | | | No clinical synovitis | | ≥12m | ✓ | - | - | - | - | - | X | - | BL & disease course & at DMARD-stop |
| Ahmad et al. (2022)[2] | AVERT-1 | | | DAS28CRP <2.6 | | 12m | ✓ | X | X | X | - | - | X | X | BL & at DMARD-stop |
| Verstappen et al. (2022)[3] | LEAC | | | No clinical synovitis | | ≥12m | ✓ | X | X | - | - | - | - | - | BL & during disease course |
| Combe et al (2021)[4] | ESPOIR | | | - | | - | - | X | X | X | - | X | X | - | BL |
| Luurssen-Masurel et al. 2021)[5] | | tREACH | | DAS44 <1.6 | | 12m | ✓ | - | - | - | - | - | - | - | - |
| Jung et al. (2020)[6] | KIMERA | | | DAS28<2.6 | | - | - | X | X | - | - | - | X | X | BL & at DMARD-stop |
| Verstappen et al. (2020)[7] | LEAC | | | No clinical synovitis | | ≥12m | ✓ | X | - | - | - | - | X | - | BL & during disease course |
| Matthijssen et al. (2020)[8] | LEAC | | | No clinical synovitis | | ≥12m | ✓ | - | - | - | - | - | X | X | BL |
| V. Mulligen et al. (2020)[9] | TARA | | | DAS44 <1.6 | | - | - | - | - | - | - | - | - | X | - |
| Manai et al. (2020)[10] | - | | | DAS44 <1.6 | | 4m | - | - | - | - | - | - | - | - | - |
| Bergstra et al. (2020)[11] | IMPROVED  BEST | | | DAS44 <1.6 | | 12m | ✓ | X | X | - | - | - | - | - | BL |
| De Moel et al. (2019)[12] | RETRO  IMPROVED | | | DAS28 <2.6  DAS44 <1.6 | | -  - | - | - | X | - | - | - | - | - | at DMARD-stop |
| Baker et al. (2019)[13] | BioRRA | | | DAS28CRP <2.4 | | - | - | X | X | X | - | X | X | - | at DMARD-stop |
| Boeters et al. (2019)[14] | LEAC | | | No clinical synovitis | | ≥12m | ✓ | - | - | - | - | - | X | - | BL & disease course & at DMARD-stop |
| Baker et al. (2019)[15] | - | | | - | | - | - | - | - | - | - | - | - | - | - |
| Boeters et al. (2019)[16] | LEAC | | | No clinical synovitis | | ≥12m | ✓ | - | X | - | - | - | - | - | BL |
| Burgers et al. (2019)[17] | LEAC  IMPROVED | | | No clinical synovitis DAS44 <1.6 | | ≥12m | ✓ | - | - | - | - | - | - | X | - |
| De Moel et al. (2019)[18] | IMPROVED | | | DAS44 <1.6 | | 12m | ✓ | - | - | - | - | - | X | - | during disease course |
| Bykerk et al. (2018)[19] | AVERT-1 | | | DAS28 <2.6 | | 6m | - | X | - | - | - | - | - | X | BL |
| De Moel et al. (2018)[20] | IMPROVED | | | DAS44 <1.6 | | 12m | ✓ | - | - | - | - | - | X | - | during disease course |
| **Author (year)** | **Cohort** | | | **Remission definition after DMARD-stop** | | **Remission**  **duration after DMARD-stop** | **SDFR** | **Clinical** | **Serology** | **Imaging** | **Histology** | **Genetics** | **Autoantibodies** | **Treatment** | **Moment markers were measured** |
| Teitsma et al. (2018)[21] | U-ACT-EARLY | | | DAS28 <2.6 | | 3m | - | - | - | - | - | X | - | - | BL |
| Teitsma et al. (2018)[22] | U-ACT-EARLY | | | DAS28 <2.6 | | 3m | - | - | - | - | - | X | - | - | BL |
| Burgers et al. (2018)[23] | LEAC | | | No clinical synovitis | | ≥12m | ✓ | X | - | - | - | - | - | - | BL |
| Akdemir et al. (2018)[24] | BEST  IMPROVED | | | DAS44 <1.6 | | - | - | X | - | - | - | - | - | X | - |
| Versteeg et al. (2018)[25] | DREAM | | | DAS28 <2.6 | | 6m | - | - | - | - | - | - | - | - | - |
| Burgers et al. (2018)[26] | LEAC | | | No clinical synovitis | | ≥12m | ✓ | - | - | X | - | - | - | - | BL |
| De Moel et al. (2018)[27] | IMPROVED | | | DAS44 <1.6 | | ≥12m | ✓ | - | - | - | - | - | X | - | BL |
| Akdemir et al. (2018)[28] | IMPROVED | | | DAS44 <1.6 | | ≥12m | ✓ | X | - | - | - | - | X | - | BL & during disease course |
| Bergstra et al. (2017)[29] | BEST | | | DAS44 <1.6 | | - | - | - | - | - | - | - | - | X | - |
| v. Hooij et al. (2017)[30] | LEAC | | | No synovitis | | ≥12m | ✓ | X | X | - | - | - | X | - | BL & during disease course |
| Teitsma et al. (2017)[31] | U-ACT-EARLY | | | DAS28 <2.6 | | 3m | - | - | - | - | - | X | - | - | BL |
| Kuijper et al. (2016)[32] | tREACH | | | DAS44 <1.6 | | - | - | X | X | X | - | - | X | - | BL |
| v. Heemst et al. (2016)[33] | LEAC | | | No synovitis | | ≥12m | ✓ | - | - | - | - | X | X | - | BL |
| Ajeganova et al. (2016)[34] | LEAC | | | No clinical synovitis | | ≥12m | ✓ | X | X | X | - | - | X | X | BL |
| Markusse et al. (2016)[35] | BEST | | | DAS44 <1.6 | | ≥12m | ✓ | - | - | - | - | - | - | - | - |
| Akdemir et al. (2016)[36] | BEST | | | DAS44 <1.6 | | - | - | - | - | - | - | - | X | - | BL |
| Heimans et al. (2016)[37] | IMPROVED | | | DAS44 <1.6 | | - | - | - | - | - | - | - | X | - | BL |
| v. Steenbergen et al. (2015)[38] | | LEAC | | No synovitis | | ≥12m | ✓ | - | - | - | - | X | - | - | BL |
| Wevers-de Boer et al. (2015)[39] | | IMPROVED | | | DAS44 <1.6 | 8m | - | X | X | X | - | - | X | - | BL |
| v. Nies et al (2015)[40] | LEAC  ESPOIR | | | No clinical synovitis | | ≥12m | ✓ | X | - | - | - | - | - | - | BL |
| v. Nies et al. (2015)[41] | LEAC | | | No clinical synovitis | | ≥12m | ✓ | X | - | - | - | - | - | - | BL |
| **Author (year)** | **Cohort** | | | **Remission definition after DMARD-stop** | | **Remission**  **duration after DMARD-stop** | **SDFR** | **Clinical** | **Serology** | **Imaging** | **Histology** | **Genetics** | **Autoantibodies** | **Treatment** | **Moment markers were measured** |
| Emery et al. (2015)[42] | AVERT-1 | | | DAS28 <2.6 | | 6m | - | X | X | X | - | - | - | X | BL |
| Huizinga et al. (2015)[43] | ACT-RAY | | | DAS28 <2.6 | | 3m | - | - | - | - | - | - | - | - | - |
| Heimans et al. (2014)[44] | IMPROVED | | | DAS44 <1.6 | | - | - | - | - | - | - | - | - | - | - |
| Markusse et al. (2014)[45] | - | | | - | | - | - | - | - | - | - | - | - | - | - |
| v. Nies et al. (2014)[46] | LEAC  ERAS  BEST | | | No clinical synovitis  No clinical synovitis DAS44 <1.6 | | ≥12m  ≥12m  ≥12m | ✓ | X | - | - | - | - | - | - | BL |
| Burgers et al. (2014)[47] | LEAC | | | No clinical synovitis | | ≥12m | ✓ | X | - | - | - | - | - | - | BL |
| Nishimoto et al. (2014)[48] | DREAM trial | | | DAS28 <3.2 | | ≥12m | ✓ | X | X | - | - | - | X | X | at DMARD-stop |
| v.d. Woude et al.(2012)[49] | LEAC  BEST | | | No clinical synovitis DAS44 <1.6 | | ≥12m | ✓ | X | X | X | - | X | X | X | BL |
| v.d. Broek et al. (2011)[50] | BEST | | | DAS44 <1.6 | | - | - | - | - | - | - | - | X | - | BL |
| Klarenbeek et al. (2011)[51] | BEST | | | DAS44 <1.6 | | ≥12m | ✓ | - | - | - | - | - | - | - | - |
| v.d. Linden et al. (2011)[52] | LEAC | | | No clinical synovitis | | ≥12m | ✓ | - | - | - | - | - | X | - | BL |
| Klarenbeek et al.(2011)[53] | BEST | | | DAS44 <1.6 | | ≥12m | ✓ | X | X | X | - | - | X | X | BL |
| v.d. Linden et al. (2010)[54] | LEAC | | | No clinical synovitis | | ≥12m | ✓ | - | - | - | - | - | X | - | BL |
| v.d. Linden et al. (2010)[55] | LEAC | | | No clinical synovitis | | ≥12m | ✓ | X | - | - | - | - | - | - | BL |
| v.d Woude et al. (2009)[56] | LEAC | | | No clinical synovitis | | ≥12m | ✓ | X | X | X | - | X | X | - | BL |
| v.d. Kooij et al. (2009)[57] | BEST | | | DAS44 <1.6 | | 11m | - | X | X | X | - | X | X | - | BL |
| **Studies found by hand search** | | | | | | | | | | | | | | | |
| **Author (year)** | **Cohort** | | | **Remission definition after DMARD-stop** | | **Remission**  **duration after DMARD-stop** | **SDFR** | **Clinical** | **Serology** | **Imaging** | **Histology** | **Genetics** | **Autoantibodies** | **Treatment** | **Moment markers**  **were measured** |
| Verstappen et al. (2022)[58] | | | LEAC  AVERT-1 | No clinical synovitis | | ≥12m | ✓ | X | - | X | - | - | X | - | BL & during disease course |
| Niemantsverdriet et al. (2019)[59] | | | LEAC  ESPOIR | No clinical synovitis | | ≥12m | ✓ | X | - | - | - | - | - | - | BL |
| El Miedany et al. (2016)[60] | - | | | DAS28 <2.6 | | ≥12m | ✓ | - | - | - | - | - | - | - | - |
| v. Steenbergen et al. (2014)[61] | | LEAC | | No clinical synovitis | | ≥12m | ✓ | - | - | - | - | X | - | - | BL |
| v. Steenbergen et al. (2015)[62] | | LEAC | | No clinical synovitis | | ≥12m | ✓ | - | X | - | - | - | - | - | BL |
| Ten Wolde et al. (1997)[35] | - | | | ARA remission | | ≥12m | ✓ | - | - | - | - | - | - | - | - |
|  |  | | |  | |  |  |  |  |  |  |  |  |  |  |

**Legend**: List of identified studies reporting on DMARD-free remission in RA. The category of markers measured in relation to DFR and the moment these were measured (baseline, during disease course, at DMARD-stop) were indicated. Studies which specifically reported on sustained DMARD-free remission (SDFR), i.e. minimally one year of sustained remission after DMARD-discontinuation, were indicated by a check mark and included in this literature review.
*BL: Baseline, DMARD: disease-modifying anti-rheumatic drugs, DAS: disease activity score, m: months*

**Supplementary S3. Baseline markers studied in relation to sustained DMARD-free remission in RA, stratified for autoantibody status**

| **Clinical markers** | **Author (year)** | **Cohort** | **Analysis method** | **Total RA population** | **Autoantibody-positive RA** | **Autoantibody-negative RA** |
| --- | --- | --- | --- | --- | --- | --- |
| **Age at baseline** | Ahmad et al. (2022)[2] | AVERT-1 (N=172) | U | - | ≠ | - |
|  | Bergstra et al (2020)[11] | BEST (N=469)  IMPROVED (N=421) | D | ≠  ≠ | -  - | -  - |
|  | Verstappen et al. (2022)[58] | LEAC (N=198)  AVERT-1 (N=174) | D | -  - | ≠  ≠ | ≠ |
|  |  |  |  |  |  | - |
|  | v. Hooij et al. (2017)[30] | LEAC (N=24) | D | ≠ | - | - |
|  | Ajeganova et al. (2016)[34] | LEAC (N=886) | D | ≠ | - | - |
|  | v.d. Woude et al. (2012)[49] | LEAC (N=424)  BEST (N=508) | U | ≠  ≠ | -  - | -  - |
|  | Klarenbeek et al. (2011)[53] | BEST (N=112) | U | ≠ | - | - |
|  | v.d Woude et al. (2009)[56] | LEAC (N=454)  ERAS (N=895) | U | ≠  ≠ | - | - |
| **Gender** | Ahmad et al. (2022)[2] | AVERT-1 (N=172) | D | - | ≠ | - |
|  | Verstappen et al. (2022)[58] | LEAC (N=198)  AVERT-1 (N=174) | D | -  - | ≠  ≠ | ≠ |
|  |  |  |  |  |  | - |
|  | Bergstra et al (2020)[11] | BEST (N=469)  IMPROVED (N=421) | D | + | -  - | -  - |
|  |  |  |  | ≠ |  |  |
|  | v. Hooij et al. (2017)[30] | LEAC (N=24) | D | ≠ | - | - |
|  | Ajeganova et al. (2016)[34] | LEAC (N=886) | D | ≠ | - | - |
|  | Nishimoto et al. (2014)[48] | DREAM trial | U | ≠ | - | - |
|  | v.d. Woude et al. (2012)[49] | LEAC (N=424)  BEST (N=508) | M | ≠ | - | - |
|  |  |  |  | + | - |  |
|  | Klarenbeek et al. (2011)[53] | BEST (N=112) | U | ≠ | - | - |
|  | v.d Woude et al. (2009)[56] | LEAC (N=454)  ERAS (N=895) | U | ≠  ≠ | - | - |
| **Onset of symptoms** | v.d Woude et al. (2009)[56] | LEAC (N=454)  ERAS (N=895) | M | ≠ | -  - | -  - |
|  |  |  |  | + |  |  |
| **Morning stiffness**  **at baseline** | Ajeganova et al. (2016)[34] | LEAC (N=886) | D | ≠ | - | - |
|  | v. Nies et al. (2015)[41] | LEAC (N=807) | M | ≠ | - | - |
| **Smoking (yes/no)** | Bergstra et al (2020)[11] | BEST (N=469)  IMPROVED (N=421) | D | ≠  ≠ | -  - | -  - |
|  | Ajeganova et al. (2016)[34] | LEAC (N=886) | D | ≠ | - | - |
|  | v.d. Woude et al. (2012)[49] | LEAC (N=424)  BEST (N=508) | M | ≠  ≠ | -  - | -  - |
|  | v.d Woude et al. (2009)[56] | LEAC (N=454)  ERAS (N=895) | M | ≠  ≠ | -  - | -  - |
| **BMI at baseline** | Ahmad et al. (2022)[2] | AVERT-1 (N=172) | D | - | ≠ | - |
|  | Bergstra et al (2020)[11] | BEST (N=469)  IMPROVED (N=421) | D | ≠  ≠ | -  - | -  - |
|  | v.d. Woude et al. (2012)[49] | LEAC (N=424)  BEST (N=508) | U | ≠  ≠ | -  - | -  - |
|  | v.d Woude et al. (2009)[56] | ERAS (N=895) | U | ≠ | - | - |
| **Symptom duration** | v. Hooij et al. (2017)[30] | LEAC (N=24) | D | + | - | - |
|  | Bergstra et al (2020)[11] | BEST (N=469)  IMPROVED (N=421) | S | + | -  - | -  - |
|  |  |  |  | + |  |  |
|  | Niemantsverdriet et al. (2019)[59] | LEAC (N=1025)  ESPOIR (N=514) | M | +  + | -  - | -  - |
|  | Ahmad et al. (2022)[2] | AVERT-1 (N=172) | U | - | ≠ | - |
|  | v.d. Linden et al. (2011)[55] | LEAC (N=598) | M | + | - | - |
|  | Verstappen et al. (2022)[58] | LEAC (N=198) | D | - | ≠ | ≠ |
|  | v. Nies et al (2015)[40] | LEAC (N=738)  ESPOIR (N=533) | M | +  + | +  + | +  + |
|  | v. Nies et al. (2014)[46] | LEAC (N=676)  ERAS (N=895)  BEST (n=508) | P | +  +  + | -  - | -  - |
|  | Klarenbeek et al. (2011)[53] | BEST (N=112) | U | ≠ | - | - |
|  | v.d. Woude et al. (2012)[49] | LEAC (N=424)  BEST (N=508) | U | + | -  - | -  - |
|  |  |  |  | ≠ |  |  |
|  | v.d Woude et al. (2009)[56] | LEAC (N=454)  ERAS (N=895) | M | +  + | -  - | -  - |
| **Disease activity scores**  **at baseline** | Verstappen et al. (2022)[58] | LEAC (N=198)  AVERT-1 (N=174) | D | -  - | +  + | ≠ |
|  |  |  |  |  |  | - |
|  | Bergstra et al (2020)[11] | BEST (N=469)  IMPROVED (N=421) | D | + | - | - |
|  |  |  |  | ≠ | - | - |
|  | Verstappen et al. (2020)[7] | LEAC (N=772) | R | ≠ | ≠ | ≠ |
|  | v.d. Woude et al. (2012)[49] | BEST (N=508) | U | + | - | - |
|  | Klarenbeek et al. (2011)[53] | BEST (N=112) | U | ≠ | - | - |
| **SJC at baseline** | Verstappen et al. (2022)[58] | LEAC (N=198)  AVERT-1 (N=174) | D | -  - | +  + | ≠ |
|  |  |  |  |  |  | - |
|  | Verstappen et al. (2020)[7] | LEAC (N=772) | R | + | - | - |
|  | Burgers et al. (2018)[23] | LEAC (N=1233) | M | + | - | - |
|  | v. Hooij et al. (2017)[30] | LEAC (N=24) | D | + | - | - |
|  | Ajeganova et al. (2016)[34] | LEAC (N=886) | D | ≠ | - | - |
|  | v.d. Woude et al. (2012)[49] | LEAC (N=424)  BEST (N=508) | U | ≠  ≠ | -  - | -  - |
|  | v.d Woude et al. (2009)[56] | LEAC (N=454)  ERAS (N=895) | M | ≠  ≠ | -  - | -  - |
|  | Klarenbeek et al. (2011)[53] | BEST (N=112) | U | ≠ | - | - |
| **TJC at baseline** | Verstappen et al. (2022)[58] | LEAC (N=198)  AVERT-1 (N=174) | **D** | -  - | ≠  ≠ | ≠ |
|  |  |  |  |  |  | - |
|  | Verstappen et al. (2020)[7] | LEAC (N=772) | R | ≠ | - | - |
|  | v. Hooij et al. (2017)[30] | LEAC (N=24) | D | + | - | - |
|  | Ajeganova et al. (2016)[34] | LEAC (N=886) | D | ≠ | - | - |
|  | Klarenbeek et al. (2011)[53] | BEST (N=112) | U | ≠ | - | - |
|  | v.d Woude et al. (2009)[56] | LEAC (N=454)  ERAS (N=895) | M | ≠ | -  - | -  - |
|  |  |  |  | + |  |  |
| **CRP-levels at baseline** | Verstappen et al. (2022)[3] | LEAC (N=266) | M | - | ≠ | + |
|  | Verstappen et al. (2022)[58] | LEAC (N=198)  AVERT-1 (N=174) | D | -  - | ≠ | ≠ |
|  |  |  |  |  | + | - |
|  | Boeters et al. (2019)[16] | LEAC (N=299) | M | - | ≠ | + |
|  | v. Hooij et al. (2017)[30] | LEAC (N=24) | D | ≠ | - | - |
|  | v.d. Woude et al. (2012)[49] | LEAC (N=424)  BEST (N=508) | U | ≠  ≠ | -  - | -  - |
|  | Klarenbeek et al. (2011)[53] | BEST (N=112) | U | ≠ | - | - |
|  | v.d Woude et al. (2009)[56] | LEAC (N=454) | M | + | - | - |
|  | Ajeganova et al. (2016)[34] | LEAC (N=886) | D | ≠ | - | - |
| **ESR at baseline** | Verstappen et al. (2020)[7] | LEAC (N=772) | R | ≠ | - | - |
|  | v. Hooij et al. (2017)[30] | LEAC (n=24) | D | + | - | - |
|  | Ajeganova et al. (2016)[34] | LEAC (N=886) | D | ≠ | - | - |
|  | v.d. Woude et al. (2012)[49] | LEAC (N=424)  BEST (N=508) | U | ≠  ≠ | -  - | -  - |
|  | Klarenbeek et al. (2011)[53] | BEST (N=112) | U | ≠ | - | - |
|  | v.d Woude et al. (2009)[56] | LEAC (N=454)  ERAS (N=895) | M | ≠  ≠ | -  - | -  - |
| **Physical functioning**  **(HAQ-DI) at baseline** | Verstappen et al. (2022)[58] | LEAC (N=198)  AVERT-1 (N=174) | D | -  - | ≠  ≠ | ≠ |
|  |  |  |  |  |  | - |
|  | Bergstra et al (2020)[11] | BEST (N=469)  IMPROVED (N=421) | D | + | - | - |
|  |  |  |  | ≠ | - | - |
|  | Ajeganova et al. (2016)[34] | LEAC (N=886) | D | ≠ | - | - |
|  | v.d. Woude et al. (2012)[49] | LEAC (N=424)  BEST (N=508) | U | ≠ | - | - |
|  |  |  |  | + | - | - |
|  | Klarenbeek et al. (2011)[53] | BEST (N=112) | M | + | - | - |
|  | v.d Woude et al. (2009)[56] | LEAC (N=454)  ERAS (N=895) | M | + | -  - | -  - |
|  |  |  |  | ≠ |  |  |
| **Fatigue at baseline** | Ajeganova et al. (2016)[34] | LEAC (N=886) | D | + |  |  |
| **Patient global assessment at baseline** | Verstappen et al. (2022)[58] | LEAC (N=198)  AVERT-1 (N=174) | D | -  - | ≠ | ≠ |
|  |  |  |  |  | + | - |
|  | Verstappen et al. (2020)[7] | LEAC (N=772) | R | ≠ | - | - |
|  | Ajeganova et al. (2016)[34] | LEAC (N=886) | D | ≠ | - | - |
|  | Klarenbeek et al. (2011)[53] | BEST (N=112) | U | + | - | - |
| **RA-criteria** | Burgers et al. (2014)[47] | LEAC (N=1338) | M | + | - | - |
|  | | | | | | |
| **Serological markers** | **Author (year)** | **Cohort** | **Analysis method** | **Total RA population** | **Autoantibody-positive RA** | **Autoantibody-negative RA** |
| **MBDA score (total)*** | Boeters et al. (2019)[16] | LEAC (N=299) | M | + | ≠ | + |
| **MMP-3** (levels at baseline) | Boeters et al. (2019)[16] | LEAC (N=299) | M | - | - | + |
|  | Verstappen et al. (2022)[3] | LEAC (N=266) | R | - | ≠ | + |
| **MMP-1** (levels at baseline) | Boeters et al. (2019)[16] | LEAC (N=299) | M | - | - | ≠ |
|  | Verstappen et al. (2022)[3] | LEAC (N=266) | R | - | ≠ | ≠ |
| **SAA** (levels at baseline) | Boeters et al. (2019)[16] | LEAC (N=299) | M | - | - | + |
|  | Verstappen et al. (2022)[3] | LEAC (N=266) | R | - | ≠ | + |
| **Il-6** (levels at baseline) | Boeters et al. (2019)[16] | LEAC (N=299) | M | - | - | ≠ |
|  | Verstappen et al. (2022)[3] | LEAC (N=266) | R | - | - | ≠ |
| **Leptin** (levels at baseline) | Boeters et al. (2019)[16] | LEAC (N=299) | M | - | - | ≠ |
|  | Verstappen et al. (2022)[3] | LEAC (N=266) | R | - | - | ≠ |
| **Resistin** (levels at baseline) | Boeters et al. (2019)[16] | LEAC (N=299) | M | - | -- | ≠ |
|  | Verstappen et al. (2022)[3] | LEAC (N=266) | R | - | - | ≠ |
| **TNF-R1** (levels at baseline) | Boeters et al. (2019)[16] | LEAC (N=299) | M | - | - | ≠ |
|  | Verstappen et al. (2022)[3] | LEAC (N=266) | R | - | - | ≠ |
| **VCAM-1** (levels at baseline) | Boeters et al. (2019)[16] | LEAC (N=299) | M | - | - | ≠ |
|  | Verstappen et al. (2022)[3] | LEAC (N=266) | R | - | - | ≠ |
| **EGF** (levels at baseline) | Boeters et al. (2019)[16] | LEAC (N=299) | M | - | - | ≠ |
|  | Verstappen et al. (2022)[3] | LEAC (N=266) | R | - | - | ≠ |
| **VEGF** (levels at baseline) | Boeters et al. (2019)[16] | LEAC (N=299) | M | - | - | ≠ |
|  | Verstappen et al. (2022)[3] | LEAC (N=266) | R | - | - | ≠ |
| **YKL-40** (levels at baseline) | Boeters et al. (2019)[16] | LEAC (N=299) | M | - | - | ≠ |
|  | Verstappen et al. (2022)[3] | LEAC (N=266) | R | - | - | ≠ |
| **IP-10** (levels at baseline) | v. Hooij et al. (2017)[30] | LEAC (N=24) | R | ≠ | - | - |
|  | | | | | | |
| **Imaging** | **Author (year)** | **Cohort** | **Analysis method** | **Total RA population** | **Autoantibody-positive RA** | **Autoantibody-negative RA** |
| **SHS** | v.d. Woude et al. (2012)[49] | LEAC (N=424)  BEST (N=508) | U | ≠  ≠ | -  - | -  - |
|  | Klarenbeek et al. (2011)[53] | BEST (N=508) | U | ≠ | - | - |
|  | v.d Woude et al. (2009)[56] | LEAC (N=454)  ERAS (N=895) | U | + | -  - | -  - |
|  |  |  |  | ≠ |  |  |
| **MRI** | Burgers et al. (2018)[26] | LEAC (N=238) | M | ≠ | - | - |
|  | Verstappen et al. (2022)[58] | LEAC (N=198)  AVERT-1 (N=174) | R | -  - | +  + | ≠ |
|  |  |  |  |  |  | - |
|  | | | | | | |
| **Genetic markers** | **Author (year)** | **Cohort** | **Analysis method** | **Total RA population** | **Autoantibody-positive RA** | **Autoantibody-negative RA** |
| **HLA shared epitope** | v.d. Woude et al. (2012)[49] | LEAC (N=424)  BEST (N=508) | U | ≠  ≠ | -  - | -  - |
|  | v.d Woude et al. (2009)[56] | LEAC (N=454)  ERAS (N=895) | M | ≠ | -  - | -  - |
|  |  |  |  | + |  |  |
|  | v. Steenbergen et al. (2015)[38] | LEAC (N=645) | M | + | ≠ | ≠ |
| **HLA-DRB1*13** | v. Heemst et al. (2016)[33] | LEAC (N=441) | M | ≠ | - | - |
| **IL2RA-rs2104286** | v. Steenbergen et al. (2015)[38] | LEAC (N=645) | M | + | ≠ | + |
| **Dickkopf-1** | v. Steenbergen et al. (2015)[38] | LEAC (N=645) | M | ≠ | - | - |
| **MMP-9** | v. Steenbergen et al. (2015)[38] | LEAC (N=645) | M | ≠ | - | - |
| **OPG** | v. Steenbergen et al. (2015)[38] | LEAC (N=645) | M | ≠ | - | - |
| **FOX03A** | v. Steenbergen et al. (2014)[61] | LEAC (N=645) | M | ≠ | - | - |
|  | | | | | | |
| **Autoantibody (characteristics)** | **Author (year)** | **Cohort** | **Analysis method** | **Total RA population** | **Autoantibody-positive RA** | **Autoantibody-negative RA** |
| **ACPA-positivity** | Matthijssen et al. (2020)[8] | LEAC (N=1285) | D | + | n.a. | n.a. |
|  | Verstappen et al. (2020)[7] | LEAC (N=772) | R | + | n.a. | n.a. |
|  | Bergstra et al (2020)[11] | BEST (N=469)  IMPROVED (N=421) | D | +  + | - | - |
|  | v. Hooij et al. (2017)[30] | LEAC (N=24) | D | + | n.a. | n.a. |
|  | Ajeganova et al. (2016)[34] | LEAC (N=886) | D | + | n.a. | n.a. |
|  | v.d. Woude et al. (2012)[49] | LEAC (N=424)  BEST (N=508) | U | ≠  ≠ | n.a. | n.a. |
|  | Klarenbeek et al. (2011)[53] | BEST (N=112) | M | + | n.a. | n.a. |
|  | v.d. Linden et al. (2010) | LEAC (N=687) | M | + | n.a. | n.a. |
|  | v.d Woude et al. (2009)[56] | LEAC (N=454) | M | + | n.a. | n.a. |
| **RF-positivity** | Verstappen et al. (2022)[58] | LEAC (N=198)  AVERT-1 (N=174) | D | -  - | ≠  ≠ | + |
|  |  |  |  |  |  | - |
|  | Bergstra et al. (2020)[11] | IMPROVED (N=479)  BEST (N=508) | U | ≠ | - | - |
|  |  |  |  | + | - | - |
|  | v. Hooij et al. (2017)[30] | LEAC (N=24) | D | ≠ | - | - |
|  | Ajeganova et al. (2016)[34] | LEAC (N=886) | D | + | - | - |
|  | Nishimoto et al. (2014)[48] | DREAM (N=187) | M | ≠ | - | - |
|  | Klarenbeek et al. (2011)[53] | BEST (N=112) | U | + | - | - |
|  | v.d Woude et al. (2009)[56] | LEAC (N=454)  ERAS (N=895) | M | ≠ | -  - | -  - |
|  |  |  |  | + |  |  |
|  | v.d. Linden et al. (2010) | LEAC (N=687) | M | + | - | - |
|  | v.d. Woude et al. (2012)[49] | LEAC (N=424)  BEST (N=508) | M | ≠  ≠ | -  - | -  - |
|  |  |  |  |  |  |  |
| **ACPA/RF-levels**  **at baseline** | v.d. Linden et al. (2011)[52] | LEAC (N=687) | M | ≠ | -  - | -  - |
|  | Boeters et al. (2019)[14] | LEAC (N=95) |  | - | ≠ | n.a. |
| **Other autoantibodies** | v. Wesemael et al. (2022) | LEAC (N=234) | M | - | ≠ | n.a. |
|  | De Moel et al. (2018)[27] | IMPROVED (N=399) | M | - | ≠ | n.a. |
|  | v.d. Linden et al. (2011)[54] | LEAC (N=687) | M | ≠ | - | - |
| **Autoantibody characteristics** | Kissel et al. (2021)[1] | LEAC (N=234) | U | - | + | n.a. |

***Legend:*** Direction of association between clinical, serological, imaging and genetic markers and sustained DMARD-free remission in the total RA-population. The last twee columns indicate whether results were studied for autoantibody-positive and autoantibody-negative RA-patients separately.

D: descriptive analysis, U: univariable analysis, M: multivariable analysis, P: pooled, R: repeated measurements analysis.

*ACPA: anticitrullinated protein antibody, HLA: human leucocyte antigen, MMP: matrix metalloproteinase, OPG: osteoprotegrin, RF: rheumatoid factor*

* In the article by Boeters et al. the MBDA score (total of 12 biomarkers; MMP-1/MMP-3/SAA/CRP/IL-6/leptin/resistin/YKL-40/TNF-R1/EGF/VEGF/VCAM) and the baseline level of each independent biomarker was therefore not studied in relation to SDFR.

**Supplementary S4. Biomarkers during the disease course studied in relation to sustained DMARD-free remission in RA, stratified for autoantibody status**

|  | **Author (year)** | **Cohort** | **Analysis method** | **Total RA population** | **Autoantibody-positive RA** | **Autoantibody-negative RA** |
| --- | --- | --- | --- | --- | --- | --- |
| **Clinical markers** | | | | | | |
| **Early clinical remission** | Verstappen et al. (2020)[7] | LEAC (N=772) | R | + | ≠ | + |
|  | Akdemir et al. (2018)[28] | IMPROVED (N=610) | M | + | - | - |
| **Serological markers** | | | | | | |
| **IP-10** | v. Hooij et al. (2017)[30] | LEAC (N=24) | R | + | - | - |
| **SAA** | Verstappen et al. (2021)[3] | LEAC (N=266) | R | - | ≠ | + |
| **MMP-1** | Verstappen et al. (2021)[3] | LEAC (N=266) | R | - | ≠ | + |
| **MMP-3** | Verstappen et al. (2021)[3] | LEAC (N=266) | R | - | ≠ | + |
| **CRP** | Verstappen et al. (2021)[3] | LEAC (N=266) | R | - | ≠ | + |
| **YKL-40** | Verstappen et al. (2021)[3] | LEAC (N=266) | R | - | - | ≠ |
| **VCAM-1** | Verstappen et al. (2021)[3] | LEAC (N=266) | R | - | - | ≠ |
| **VEGF** | Verstappen et al. (2021)[3] | LEAC (N=266) | R | - | - | ≠ |
| **EGF** | Verstappen et al. (2021)[3] | LEAC (N=266) | R | - | - | ≠ |
| **Leptin** | Verstappen et al. (2021)[3] | LEAC (N=266) | R | - | - | ≠ |
| **Resistin** | Verstappen et al. (2021)[3] | LEAC (N=266) | R | - | - | ≠ |
| **IL-6** | Verstappen et al. (2021)[3] | LEAC (N=266) | R | - | - | ≠ |
| **TNF-R1** | Verstappen et al. (2021)[3] | LEAC (N=266) | R | - | - | ≠ |
| **Imaging** | | | | | | |
| **MRI** | Verstappen et al. (2022)[58] | LEAC (N=198)  AVERT-1 (N=174) | R | - | ≠ | + |
|  |  |  |  |  | ≠ | - |
| **Auto-antibodies** | | | | | | |
| **RF-levels over time** | Boeters et al. (2019)[14] | LEAC (N=941) | M | - | + | - |
|  | De Moel et al. (2018)[27] | IMPROVED (N=381) | R | - | ≠ | - |
| **ACPA-levels over time** | Boeters et al. (2019)[14] | LEAC (N=941) | M | - | ≠ | n.a. |
|  | De Moel et al. (2018)[27] | IMPROVED (N=381) | R | - | ≠ | n.a. |
| **Other autoantibody levels over time** | De Moel et al. (2018)[27]  (antiCarP/anti-acelytated lysine) | IMPROVED (N=381) | R | - | ≠ | n.a. |
| **Seroreversion** | Boeters et al. (2019)[14] (ACPA/RF) | LEAC (N=941) | M | - | ≠ | n.a. |
|  | De Moel et al. (2019)[18] | IMPROVED (N=381) | D | - | ≠ | n.a. |

**Legend:** Direction of association between clinical, serological, imaging and genetic markers during the disease course and sustained DMARD-free remission in the total RA-population. The last twee columns indicate whether results were studied for autoantibody-positive and autoantibody-negative RA-patients separately. D: descriptive analysis, U: univariable analysis, M: multivariable analysis, P: pooled, R: repeated measurements analysis.

*AB: autoantibody, HLA: human leucocyte antigen, MMP: matrix metalloproteinase*

**Supplementary S5. Biomarkers at time of DMARD-discontinuation studied in relation to sustained DMARD-free remission in RA, stratified for autoantibody status**

|  | **Author (year)** | **Cohort** | **Analysis method** | **Total RA population** | **Autoantibody-positive RA** | **Autoantibody-negative RA** |
| --- | --- | --- | --- | --- | --- | --- |
| **Clinical markers** | | | | | | |
| ***Disease duration till DMARD-stop*** | Nishimoto et al. (2014)[48] | DREAM trial (N=187) | U | ≠ | - | - |
| ***Disease activity scores at DMARD-stop*** | Nishimoto et al. (2014)[48] | DREAM trial (N=187) | M | ≠ | - | - |
|  | Ahmad et al. (2022)[2] | AVERT-1 (N=174) | M | - | ≠ | - |
|  | Klarenbeek et al. (2011)[53] | BEST (N=112) | U | ≠ | - | - |
| ***SJC at DMARD-stop*** | Ahmad et al. (2022)[2] | AVERT-1 (N=174) | U | - | ≠ | - |
|  | Klarenbeek et al. (2011)[53] | BEST (N=112) | U | ≠ | - | - |
| ***TJC at DMARD-stop*** | Ahmad et al. (2022)[2] | AVERT-1 (N=174) | U | - | ≠ | - |
|  | Klarenbeek et al. (2011)[53] | BEST (N=112) | U | ≠ | - | - |
| ***Physical functioning at DMARD-stop*** | Nishimoto et al. (2014)[48] | DREAM trial (N=187) | M | ≠ | - | - |
|  | Ahmad et al. (2022)[2] | AVERT-1 (N=174) | M | + | + | - |
|  | Klarenbeek et al. (2011)[53] | BEST (N=112) | U | ≠ | - | - |
| ***PGA at DMARD-stop*** | Ahmad et al. (2022)[2] | AVERT-1 (N=174) | M | - | ≠ | - |
|  | Klarenbeek et al. (2011)[53] | BEST (N=112) | U | ≠ | - | - |
| ***CRP-levels at DMARD-stop*** | Ahmad et al. (2022)[2] | AVERT-1 (N=174) | U | - | ≠ | - |
|  | Klarenbeek et al. (2011)[53] | BEST (N=112) | U | ≠ | - | - |
| ***ESR at DMARD-stop*** | Klarenbeek et al. (2011)[53] | BEST (N=112) | U | ≠ | - | - |
| **Serological markers** | | | | | | |
| ***IL-6*** | Nishimoto et al. (2014)[48] | DREAM trial (N=187) | M | + | - | - |
| ***MMP-3*** | Nishimoto et al. (2014)[48] | DREAM trial (N=187) | M | + | - | - |
| **Imaging** | | | | | | |
| ***X-rays*** | Nishimoto et al. (2014)[48] | DREAM trial (N=187) | M | ≠ | - | - |
|  | Klarenbeek et al. (2011)[53] | BEST (N=508) | U | ≠ | - | - |
| ***MRI*** | Ahmad et al. (2022)[2] | AVERT-1 (N=174) | M | - | + |  |
| **Auto-antibodies** | | | | | | |
| ***Seroreversion*** | Ahmad et al. (2022)[2] | AVERT-1 (N=174) | D | - | ≠ | n.a. |
|  | Boeters et al. (2019)[14] (ACPA/RF) | LEAC (N=941) | S | - | ≠ | n.a. |
|  | De Moel et al. (2019)[18] | IMPROVED (N=381) | D | - | ≠ | n.a. |

**Legend:** Direction of association between clinical, serological, imaging and genetic markers at time of DMARD-discontinuation and sustained DMARD-free remission in the total RA-population. The last twee columns indicate whether results were studied for autoantibody-positive and autoantibody-negative RA-patients separately. D: descriptive analysis, U: univariable analysis, M: multivariable analysis, P: pooled, R: repeated measurements analysis.

*AB: autoantibody, HLA: human leucocyte antigen, MMP: matrix metalloproteinase*

**Supplementary S6. Treatment strategies studied in relation to sustained DMARD-free remission in RA, stratified for autoantibody status**

|  | **Author (year)** | **Cohort** | **Analysis method** | **Total RA population** | **Autoantibody-positive RA** | **Autoantibody-negative RA** |
| --- | --- | --- | --- | --- | --- | --- |
| **Combination therapy vs monotherapy** | Ahmad et al. (2022)[2] | AVERT-1 (N=174) | D | ≠ | ≠ | - |
|  | Bergstra et al. (2020)[11] | BEST (N=508) | D | ≠ | - | - |
|  | Klarenbeek et al. (2011)[53] | BEST (N=508) | U | ≠ | **-** | **-** |
| **Early and intensive DMARD-treatment (compared to less intensive treatment)** | Ajeganova et al. (2016)[34] | LEAC (N=886) | M | + | + | + |
|  | Matthijssen et al. (2020)[8] | LEAC (N=1285) | M | + | + | ≠ |
|  | Burgers et al. (2019) | IMPROVED (N=155)  LEAC (N=124) | M | ≠ | + | ≠ |
|  | v.d. Woude et al. (2012)[49] | LEAC (N=424)  BEST (N=508) | M | ≠ | + | ≠ |

**Legend:** Direction of association between clinical, serological, imaging and genetic markers during the disease course and sustained DMARD-free remission in the total RA-population. The last twee columns indicate whether results were studied for autoantibody-positive and autoantibody-negative RA-patients separately. D: descriptive analysis, U: univariable analysis, M: multivariable analysis, P: pooled, R: repeated measurements analysis, S: survival analysis.

*AB: autoantibody, HLA: human leucocyte antigen, MMP: matrix metalloproteinase*

**REFERENCES**

[1] Kissel T, Hafkenscheid L, Wesemael TJ, Tamai M, Kawashiri SY, Kawakami A, et al. ACPA-IgG variable domain glycosylation increases before the onset of rheumatoid arthritis and stabilizes thereafter; a cross-sectional study encompassing over 1500 samples. bioRxiv 2021:2021.11.05.467407.

[2] Ahmad HA, Baker JF, Conaghan PG, Emery P, Huizinga TWJ, Elbez Y, et al. Prediction of flare following remission and treatment withdrawal in early rheumatoid arthritis: post hoc analysis of a phase IIIb trial with abatacept. Arthritis Res Ther 2022;24(1):47.

[3] Verstappen M, van Steenbergen HW, de Jong PHP, van der Helm-van Mil AHM. Unraveling heterogeneity within ACPA-negative rheumatoid arthritis: the subgroup of patients with a strong clinical and serological response to initiation of DMARD treatment favor disease resolution. Arthritis Res Ther 2022;24(1):4.

[4] Combe B, Rincheval N, Berenbaum F, Boumier P, Cantagrel A, Dieude P, et al. Current favourable 10-year outcome of patients with early rheumatoid arthritis: data from the ESPOIR cohort. Rheumatology (Oxford) 2021.

[5] Luurssen-Masurel N, van Mulligen E, Weel-Koenders A, Hazes JMW, de Jong PHP. The susceptibility of attaining and maintaining DMARD-free remission in different (rheumatoid) arthritis phenotypes. Rheumatology (Oxford) 2021.

[6] Jung SM, Pyo JY, Lee SW, Song JJ, Lee SK, Park YB. Clinical characteristics associated with drug-free sustained remission in patients with rheumatoid arthritis: Data from Korean Intensive Management of Early Rheumatoid Arthritis (KIMERA). Semin Arthritis Rheum 2020;50(6):1414-20.

[7] Verstappen M, Niemantsverdriet E, Matthijssen XME, le Cessie S, van der Helm-van Mil AHM. Early DAS response after DMARD-start increases probability of achieving sustained DMARD-free remission in rheumatoid arthritis. Arthritis Res Ther 2020;22(1):276.

[8] Matthijssen XME, Niemantsverdriet E, Huizinga TWJ, van der Helm-van Mil AHM. Enhanced treatment strategies and distinct disease outcomes among autoantibody-positive and -negative rheumatoid arthritis patients over 25 years: A longitudinal cohort study in the Netherlands. PLoS Med 2020;17(9):e1003296.

[9] van Mulligen E, Weel AE, Hazes JM, van der Helm-van Mil A, de Jong PHP. Tapering towards DMARD-free remission in established rheumatoid arthritis: 2-year results of the TARA trial. Ann Rheum Dis 2020;79(9):1174-81.

[10] Manaï M, van Middendorp H, Veldhuijzen DS, van der Pol JA, Huizinga TWJ, Evers AWM. Pharmacological conditioning in the treatment of recent-onset rheumatoid arthritis: a randomized controlled trial study protocol. Trials 2020;21(1):15.

[11] Bergstra SA, Van Der Pol JA, Riyazi N, Goekoop-Ruiterman YPM, Kerstens P, Lems W, et al. Earlier is better when treating rheumatoid arthritis: but can we detect a window of opportunity? RMD Open 2020;6(1).

[12] de Moel EC, Rech J, Mahler M, Roth J, Vogl T, Schouffoer A, et al. Circulating calprotectin (S100A8/A9) is higher in rheumatoid arthritis patients that relapse within 12 months of tapering anti-rheumatic drugs. Arthritis Res Ther 2019;21(1):268.

[13] Baker KF, Skelton AJ, Lendrem DW, Scadeng A, Thompson B, Pratt AG, et al. Predicting drug-free remission in rheumatoid arthritis: A prospective interventional cohort study. J Autoimmun 2019;105:102298.

[14] Boeters DM, Burgers LE, Toes RE, van der Helm-van Mil A. Does immunological remission, defined as disappearance of autoantibodies, occur with current treatment strategies? A long-term follow-up study in rheumatoid arthritis patients who achieved sustained DMARD-free status. Ann Rheum Dis 2019;78(11):1497-504.

[15] Baker KF, Isaacs JD, Thompson B. "Living a normal life": a qualitative study of patients' views of medication withdrawal in rheumatoid arthritis. BMC Rheumatol 2019;3:2.

[16] Boeters DM, Burgers LE, Sasso EH, Huizinga TWJ, van der Helm-van Mil AHM. ACPA-negative RA consists of subgroups: patients with high likelihood of achieving sustained DMARD-free remission can be identified by serological markers at disease presentation. Arthritis Res Ther 2019;21(1):121.

[17] Burgers LE, van der Pol JA, Huizinga TWJ, Allaart CF, van der Helm-van Mil AHM. Does treatment strategy influence the ability to achieve and sustain DMARD-free remission in patients with RA? Results of an observational study comparing an intensified DAS-steered treatment strategy with treat to target in routine care. Arthritis Res Ther 2019;21(1):115.

[18] de Moel EC, Derksen V, Trouw LA, Bang H, Collée G, Lard LR, et al. In rheumatoid arthritis, changes in autoantibody levels reflect intensity of immunosuppression, not subsequent treatment response. Arthritis Res Ther 2019;21(1):28.

[19] Bykerk VP, Burmester GR, Combe BG, Furst DE, Huizinga TWJ, Ahmad HA, et al. On-drug and drug-free remission by baseline symptom duration: abatacept with methotrexate in patients with early rheumatoid arthritis. Rheumatol Int 2018;38(12):2225-31.

[20] de Moel EC, Derksen V, Trouw LA, Bang H, Goekoop-Ruiterman YPM, Steup-Beekman GM, et al. In RA, becoming seronegative over the first year of treatment does not translate to better chances of drug-free remission. Ann Rheum Dis 2018;77(12):1836-8.

[21] Teitsma XM, Jacobs JWG, Concepcion AN, Pethö-Schramm A, Borm MEA, van Laar JM, et al. Explorative analyses of protein biomarkers in patients with early rheumatoid arthritis achieving sustained drug-free remission after treatment with tocilizumab- or methotrexate-based strategies: from transcriptomics to proteomics. Clin Exp Rheumatol 2018;36(6):976-83.

[22] Teitsma XM, Yang W, Jacobs JWG, Pethö-Schramm A, Borm MEA, Harms AC, et al. Baseline metabolic profiles of early rheumatoid arthritis patients achieving sustained drug-free remission after initiating treat-to-target tocilizumab, methotrexate, or the combination: insights from systems biology. Arthritis Res Ther 2018;20(1):230.

[23] Burgers LE, Boeters DM, van der Helm-van Mil AH. Large joint involvement at first presentation with RA, an unfavourable feature: results of a large longitudinal study with functioning and DMARD-free sustained remission as outcomes. Ann Rheum Dis 2018;77(6):e33.

[24] Akdemir G, Markusse IM, Bergstra SA, Goekoop RJ, Molenaar ET, van Groenendael J, et al. Comparison between low disease activity or DAS remission as treatment target in patients with early active rheumatoid arthritis. RMD Open 2018;4(1):e000649.

[25] Versteeg GA, Steunebrink LMM, Vonkeman HE, Ten Klooster PM, van der Bijl AE, van de Laar M. Long-term disease and patient-reported outcomes of a continuous treat-to-target approach in patients with early rheumatoid arthritis in daily clinical practice. Clin Rheumatol 2018;37(5):1189-97.

[26] Burgers LE, Boeters DM, Reijnierse M, van der Helm-van Mil AHM. Does the presence of magnetic resonance imaging-detected osteitis at diagnosis with rheumatoid arthritis lower the risk for achieving disease-modifying antirheumatic drug-free sustained remission: results of a longitudinal study. Arthritis Res Ther 2018;20(1):68.

[27] de Moel EC, Derksen V, Stoeken G, Trouw LA, Bang H, Goekoop RJ, et al. Baseline autoantibody profile in rheumatoid arthritis is associated with early treatment response but not long-term outcomes. Arthritis Res Ther 2018;20(1):33.

[28] Akdemir G, Heimans L, Bergstra SA, Goekoop RJ, van Oosterhout M, van Groenendael J, et al. Clinical and radiological outcomes of 5-year drug-free remission-steered treatment in patients with early arthritis: IMPROVED study. Ann Rheum Dis 2018;77(1):111-8.

[29] Bergstra SA, Landewé RBM, Huizinga TWJ, Allaart CF. Rheumatoid arthritis patients with continued low disease activity have similar outcomes over 10 years, regardless of initial therapy. Rheumatology (Oxford) 2017;56(10):1721-8.

[30] van Hooij A, Boeters DM, Tjon Kon Fat EM, van den Eeden SJF, Corstjens P, van der Helm-van Mil AHM, et al. Longitudinal IP-10 Serum Levels Are Associated with the Course of Disease Activity and Remission in Patients with Rheumatoid Arthritis. Clin Vaccine Immunol 2017;24(8).

[31] Teitsma XM, Jacobs JWG, Mokry M, Borm MEA, Pethö-Schramm A, van Laar JM, et al. Identification of differential co-expressed gene networks in early rheumatoid arthritis achieving sustained drug-free remission after treatment with a tocilizumab-based or methotrexate-based strategy. Arthritis Res Ther 2017;19(1):170.

[32] Kuijper TM, Luime JJ, de Jong PH, Gerards AH, van Zeben D, Tchetverikov I, et al. Tapering conventional synthetic DMARDs in patients with early arthritis in sustained remission: 2-year follow-up of the tREACH trial. Ann Rheum Dis 2016;75(12):2119-23.

[33] van Heemst J, Hensvold AH, Jiang X, van Steenbergen H, Klareskog L, Huizinga TW, et al. Protective effect of HLA-DRB1*13 alleles during specific phases in the development of ACPA-positive RA. Ann Rheum Dis 2016;75(10):1891-8.

[34] Ajeganova S, van Steenbergen HW, van Nies JA, Burgers LE, Huizinga TW, van der Helm-van Mil AH. Disease-modifying antirheumatic drug-free sustained remission in rheumatoid arthritis: an increasingly achievable outcome with subsidence of disease symptoms. Ann Rheum Dis 2016;75(5):867-73.

[35] Markusse IM, Akdemir G, Dirven L, Goekoop-Ruiterman YP, van Groenendael JH, Han KH, et al. Long-Term Outcomes of Patients With Recent-Onset Rheumatoid Arthritis After 10 Years of Tight Controlled Treatment: A Randomized Trial. Ann Intern Med 2016;164(8):523-31.

[36] Akdemir G, Markusse IM, Dirven L, Riyazi N, Steup-Beekman GM, Kerstens P, et al. Effectiveness of four dynamic treatment strategies in patients with anticitrullinated protein antibody-negative rheumatoid arthritis: a randomised trial. RMD Open 2016;2(1):e000143.

[37] Heimans L, Akdemir G, Boer KV, Goekoop-Ruiterman YP, Molenaar ET, van Groenendael JH, et al. Two-year results of disease activity score (DAS)-remission-steered treatment strategies aiming at drug-free remission in early arthritis patients (the IMPROVED-study). Arthritis Res Ther 2016;18:23.

[38] van Steenbergen HW, van Nies JA, Ruyssen-Witrand A, Huizinga TW, Cantagrel A, Berenbaum F, et al. IL2RA is associated with persistence of rheumatoid arthritis. Arthritis Res Ther 2015;17(1):244.

[39] Wevers-de Boer KV, Heimans L, Visser K, Schouffoer AA, Molenaar ET, van Groenendael JH, et al. Determinants of reaching drug-free remission in patients with early rheumatoid or undifferentiated arthritis after one year of remission-steered treatment. Rheumatology (Oxford) 2015;54(8):1380-4.

[40] van Nies JA, Tsonaka R, Gaujoux-Viala C, Fautrel B, van der Helm-van Mil AH. Evaluating relationships between symptom duration and persistence of rheumatoid arthritis: does a window of opportunity exist? Results on the Leiden early arthritis clinic and ESPOIR cohorts. Ann Rheum Dis 2015;74(5):806-12.

[41] van Nies JA, Alves C, Radix-Bloemen AL, Gaujoux-Viala C, Huizinga TW, Hazes JM, et al. Reappraisal of the diagnostic and prognostic value of morning stiffness in arthralgia and early arthritis: results from the Groningen EARC, Leiden EARC, ESPOIR, Leiden EAC and REACH. Arthritis Res Ther 2015;17(1):108.

[42] Emery P, Burmester GR, Bykerk VP, Combe BG, Furst DE, Barré E, et al. Evaluating drug-free remission with abatacept in early rheumatoid arthritis: results from the phase 3b, multicentre, randomised, active-controlled AVERT study of 24 months, with a 12-month, double-blind treatment period. Ann Rheum Dis 2015;74(1):19-26.

[43] Huizinga TW, Conaghan PG, Martin-Mola E, Schett G, Amital H, Xavier RM, et al. Clinical and radiographic outcomes at 2 years and the effect of tocilizumab discontinuation following sustained remission in the second and third year of the ACT-RAY study. Ann Rheum Dis 2015;74(1):35-43.

[44] Heimans L, Wevers-de Boer KV, Visser K, Goekoop RJ, van Oosterhout M, Harbers JB, et al. A two-step treatment strategy trial in patients with early arthritis aimed at achieving remission: the IMPROVED study. Ann Rheum Dis 2014;73(7):1356-61.

[45] Markusse IM, Akdemir G, Huizinga TW, Allaart CF. Drug-free holiday in patients with rheumatoid arthritis: a qualitative study to explore patients' opinion. Clin Rheumatol 2014;33(8):1155-9.

[46] van Nies JA, Krabben A, Schoones JW, Huizinga TW, Kloppenburg M, van der Helm-van Mil AH. What is the evidence for the presence of a therapeutic window of opportunity in rheumatoid arthritis? A systematic literature review. Ann Rheum Dis 2014;73(5):861-70.

[47] Burgers LE, van Nies JA, Ho LY, de Rooy DP, Huizinga TW, van der Helm-van Mil AH. Long-term outcome of rheumatoid arthritis defined according to the 2010-classification criteria. Ann Rheum Dis 2014;73(2):428-32.

[48] Nishimoto N, Amano K, Hirabayashi Y, Horiuchi T, Ishii T, Iwahashi M, et al. Drug free REmission/low disease activity after cessation of tocilizumab (Actemra) Monotherapy (DREAM) study. Mod Rheumatol 2014;24(1):17-25.

[49] van der Woude D, Visser K, Klarenbeek NB, Ronday HK, Peeters AJ, Kerstens PJ, et al. Sustained drug-free remission in rheumatoid arthritis after DAS-driven or non-DAS-driven therapy: a comparison of two cohort studies. Rheumatology (Oxford) 2012;51(6):1120-8.

[50] van den Broek M, Dirven L, Klarenbeek NB, Molenaar TH, Han KH, Kerstens PJ, et al. The association of treatment response and joint damage with ACPA-status in recent-onset RA: a subanalysis of the 8-year follow-up of the BeSt study. Ann Rheum Dis 2012;71(2):245-8.

[51] Klarenbeek NB, Güler-Yüksel M, van der Kooij SM, Han KH, Ronday HK, Kerstens PJ, et al. The impact of four dynamic, goal-steered treatment strategies on the 5-year outcomes of rheumatoid arthritis patients in the BeSt study. Ann Rheum Dis 2011;70(6):1039-46.

[52] van der Linden MP, Batstra MR, Bakker-Jonges LE, Detert J, Bastian H, Scherer HU, et al. Toward a data-driven evaluation of the 2010 American College of Rheumatology/European League Against Rheumatism criteria for rheumatoid arthritis: is it sensible to look at levels of rheumatoid factor? Arthritis Rheum 2011;63(5):1190-9.

[53] Klarenbeek NB, van der Kooij SM, Güler-Yüksel M, van Groenendael JH, Han KH, Kerstens PJ, et al. Discontinuing treatment in patients with rheumatoid arthritis in sustained clinical remission: exploratory analyses from the BeSt study. Ann Rheum Dis 2011;70(2):315-9.

[54] van der Linden MP, van der Woude D, Ioan-Facsinay A, Levarht EW, Stoeken-Rijsbergen G, Huizinga TW, et al. Value of anti-modified citrullinated vimentin and third-generation anti-cyclic citrullinated peptide compared with second-generation anti-cyclic citrullinated peptide and rheumatoid factor in predicting disease outcome in undifferentiated arthritis and rheumatoid arthritis. Arthritis Rheum 2009;60(8):2232-41.

[55] van der Linden MP, le Cessie S, Raza K, van der Woude D, Knevel R, Huizinga TW, et al. Long-term impact of delay in assessment of patients with early arthritis. Arthritis Rheum 2010;62(12):3537-46.

[56] van der Woude D, Young A, Jayakumar K, Mertens BJ, Toes RE, van der Heijde D, et al. Prevalence of and predictive factors for sustained disease-modifying antirheumatic drug-free remission in rheumatoid arthritis: results from two large early arthritis cohorts. Arthritis Rheum 2009;60(8):2262-71.

[57] van der Kooij SM, Goekoop-Ruiterman YP, de Vries-Bouwstra JK, Güler-Yüksel M, Zwinderman AH, Kerstens PJ, et al. Drug-free remission, functioning and radiographic damage after 4 years of response-driven treatment in patients with recent-onset rheumatoid arthritis. Ann Rheum Dis 2009;68(6):914-21.

[58] Verstappen M, Matthijssen XME, Connolly SE, Maldonado MA, Huizinga TWJ, van der Helm-van Mil AHM. ACPA-negative and ACPA-positive RA patients achieving disease resolution demonstrate distinct patterns of MRI-detected joint-inflammation. Rheumatology 2022.

[59] Niemantsverdriet E, Dougados M, Combe B, van der Helm-van Mil AHM. Referring early arthritis patients within 6 weeks versus 12 weeks after symptom onset: an observational cohort study. The Lancet Rheumatology 2020;2(6):e332-e8.

[60] El Miedany Y, El Gaafary M, Youssef S, Ahmed I, Bahlas S, Hegazi M, et al. Optimizing therapy in inflammatory arthritis: prediction of relapse after tapering or stopping treatment for rheumatoid arthritis patients achieving clinical and radiological remission. Clin Rheumatol 2016;35(12):2915-23.

[61] van Steenbergen HW, Rantapää-Dahlqvist S, van Nies JA, Berglin E, Huizinga TW, Gregersen PK, et al. Does a genetic variant in FOXO3A predict a milder course of rheumatoid arthritis? Arthritis Rheumatol 2014;66(6):1678-81.

[62] van Steenbergen HW, van der Helm-van Mil AHM. Osteoprotegerin as biomarker for persistence of rheumatoid arthritis. Rheumatology 2015;55(5):949-50.
